# Hospitalizations Due to Adverse Drug Events in the Elderly—A Retrospective Register Study

**DOI:** 10.3389/fphar.2016.00358

**Published:** 2016-10-05

**Authors:** Outi Laatikainen, Sami Sneck, Risto Bloigu, Minna Lahtinen, Timo Lauri, Miia Turpeinen

**Affiliations:** ^1^Research Unit of Biomedicine and Medical Research Center Oulu, University of OuluOulu, Finland; ^2^Administration Center, Oulu University HospitalOulu, Finland; ^3^Medical Informatics Group, University of OuluOulu, Finland; ^4^Department of Internal Medicine, Oulu University HospitalOulu, Finland

**Keywords:** adverse drug events, adverse drug reactions, drug-drug interactions, elderly, polypharmacy, hospitalization

## Abstract

Adverse drug events (ADEs) are more likely to affect geriatric patients due to physiological changes occurring with aging. Even though this is an internationally recognized problem, similar research data in Finland is still lacking. The aim of this study was to determine the number of geriatric medication-related hospitalizations in the Finnish patient population and to discover the potential means of recognizing patients particularly at risk of ADEs. The study was conducted retrospectively from the 2014 emergency department patient records in Oulu University Hospital. A total number of 290 admissions were screened for ADEs, adverse drug reactions (ADRs) and drug-drug interactions (DDIs) by a multi-disciplinary research team. Customized Naranjo scale was used as a control method. All admissions were categorized into “probable,” “possible,” or “doubtful” by both assessment methods. In total, 23.1% of admissions were categorized as “probably” or “possibly” medication-related. Vertigo, falling, and fractures formed the largest group of ADEs. The most common ADEs were related to medicines from N class of the ATC-code system. Age, sex, residence, or specialty did not increase the risk for medication-related admission significantly (min *p* = 0.077). Polypharmacy was, however, found to increase the risk (OR 3.3; 95% CI, 1.5–6.9; *p* = 0.01). In conclusion, screening patients for specific demographics or symptoms would not significantly improve the recognition of ADEs. In addition, as ADE detection today is largely based on voluntary reporting systems and retrospective manual tracking of errors, it is evident that more effective methods for ADE detection are needed in the future.

## Introduction

Medicating geriatric patients is a process that requires more thought and planning than medicating younger adults. The reason for this lies partly in the physiological changes that occur with aging, e.g., changes in the body mass distribution, renal function, metabolic capacity, and alterations in blood protein levels (Mangoni and Jackson, 2003; Notenboom et al., 2014). Similar changes often increase morbidity in the aged population by weakening homeostasis but also change the pharmacodynamics and pharmacokinetics of many drugs. In addition, the inter-individual variation in these physiological changes increases with age. Therefore, it can be a challenge to predict what effect even a common medicine can have in a geriatric patient. On the other hand, weakening cognition and other practical problems can cause unwanted results in pharmacotherapy through unintended mishaps (Mangoni and Jackson, 2003; Notenboom et al., 2014). There is also an evident lack in the information on drug use in the geriatric patient population.

Altered drug pharmacokinetics and pharmacodynamics, frailty, multiple comorbidities, and simultaneous use of multiple medicines (i.e., polypharmacy) result in an increased risk for drug-drug interactions (DDIs), adverse drug reactions (ADRs), and adverse drug events (ADEs) in the geriatric population (Mangoni and Jackson, 2003; Alwahassi et al., 2014; Bérnard-Labière et al., 2015). It has been estimated that the risk of ADR is 4 times higher in the elderly than the rest of the population (Beijer and de Blaey, 2002). DDIs, ADRs, and ADEs are known to weaken the quality of life and increase the risk for morbidity and mortality (Onder et al., 2002; Hohl et al., 2013; Gutherie et al., 2015). Understandably, they are also a common cause of geriatric hospitalizations.

The aim of this study was to (1) determine the number of patients admitted to Oulu University Hospital emergency department due to ADEs, and (2) to observe if we could find leads to the potential means of recognizing patients with medication-related admission from larger patient populations.

## Materials and methods

### Study design

**Figure 1:**
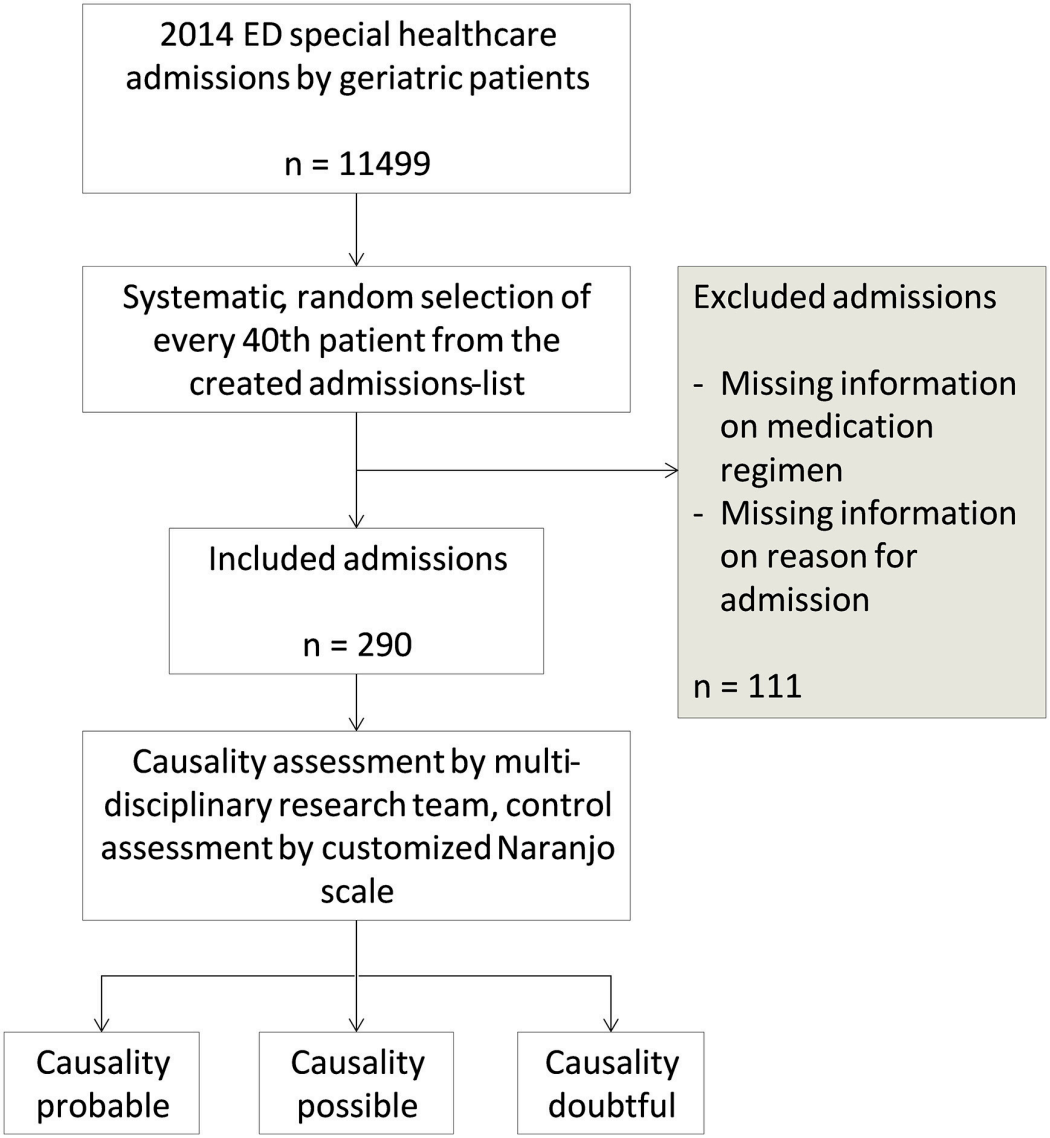
**Study design**.

This was a retrospective study in which data was collected from the 2014 emergency department (ED) patient records from a tertiary care teaching hospital, Oulu University Hospital, in Oulu, Finland. In 2014, there were 11499 ED special healthcare admissions to Oulu University Hospital by geriatric patients. From these admissions, 290 (2.5%) cases were selected for this study by a systematic random selection (Figure 1). All included admissions were made by patients aged 65 or over and treated in special healthcare unit. Patients admitted to ED nurse's reception or ED primary healthcare unit were not included in this study.

Medication record screening for potential ADEs related to hospital admissions was performed by a multi-disciplinary team including a pharmacist, a clinical pharmacologist, and a health science researcher. Databases, such as the Swedish, Finnish Interaction X-referencing (SFINX), Pharmacological Assessment on-line (PHARAO), and the geriatric medicine database created by Finnish Medicine Agency were used to detect DDIs, and potential ADRs, and ADEs. Although the causality assessment conducted by the research team was considered the final assessment, the customized Naranjo scale was also applied as control method (Table 1). The use of patient record data for this study was granted by the Oulu University Hospital's medical director and approved by the Regional Ethics Committee of the Northern Ostrobothnia Hospital District.

**Table 1:** **Assessment criteria used by the research team and the customized Naranjo scale**.

	**Expert panel**	**Customized Naranjo scale**
Probable	• Plausible relationship to drug intake	Points 5–8
	• Definite pharmacological or phenomenological explanation	
	• Definite laboratory results indicating ADR/drug interaction	
	• Drug recently added to medication regimen	
Possible	• Plausible relationship to drug intake	Points 1–4
	• Definite pharmacological or phenomenological explanation	
Doubtful	• No definite pharmacological or phenomenological explanation	Points ≤ 0

The World health Organization (WHO) defines ADRs as “noxious or unintended response to drug occurring at doses normally used in man for the prophylaxis, diagnosis or therapy of disease, or for modification of physiological function” (World Health Organization, 2002). ADE is defined as an untoward or unintended event that occurs while patient is taking a drug, but is not necessarily directly caused by the drug. According to this definition ADEs can occur as untoward or unintended injuries, symptoms, signs or abnormal laboratory values arising from appropriate or inappropriate use of medication (Edwards and Aronson, 2000; Hohl et al., 2013). ADEs therefore include reactions directly linked to drug intake (ADR, DDI) and indirect untoward events attributable to medication use (Stausberg, 2014). Medication-related hospitalization is defined as ED admission caused by an ADE. Admissions for the treatment of attempted suicide or suboptimal medication were not considered medication-related. Polypharmacy was defined as simultaneous use of multiple drugs (World Health Organization, 2004). Although there is no distinct number of medications agreed upon to define polypharmacy, it is commonly characterized as concomitant use of 5 or more drugs (World Health Organization, 2004; Gutherie et al., 2015).

### Data collection

The 290 ED admission cases selected for this study were achieved by assigning each 2014 special healthcare admission a number (1–11,499) and including every fortieth case. The systematic randomization method used was applied to obtain a sample that had little or no bias due to seasonal and population variables. Sample selection method allowed one patient to appear multiple times in the sample due to separate admissions to the ED. If information on medication regimen or reason for admission was lacking, the case was excluded from the sample and replaced by the next ED admission on the created admissions-list. On this basis, 111 admissions were excluded from the sample. No other exclusion criteria were used. All data was anonymized.

Included admissions were tested for bias toward the selected variables (age, sex, specialty, month of ED admission). No bias toward any of these variables were detected (min *p* = 0.630). However, it was noted that a relatively larger amount of surgical patients and a smaller amount of internal medicine patients were excluded than expected. In addition, the amount of included surgical patients was smaller and the amount of internal medicine patients was higher than expected. This was not considered to be caused by faulty sampling, but rather by expressing differences in documenting medication information amongst different specialties.

Electronic patient records for each included ED visit were reviewed. Information on patients' demographics (age, sex, living arrangement), comorbidities, medication regimen, ADE, drug interactions, and reason for admission was gathered. When relevant, information was supplemented with the patient's laboratory results. Admissions were divided into groups according to different sociodemographic variables for further analysis (Table 2).

**Table 2:** **Sociodemographic details of the study sample (*n* = 290)**.

**Parameter**		**Medication related admission**	
		**Yes (*n* = 67)**	**No (*n* = 223)**
Gender [n (%)]	Male	27 (18.9)	116 (81.1)
	Female	40 (27.2)	107 (72.8)
Age [n (%)]	65–74 y	21 (19.3)	88 (80.7)
	75–84 y	29 (21.8)	104 (78.2)
	85–95 y	17 (35.4)	31 (64.6)
Comorbidities [n (%)]	0	2 (9.1)	20 (90.9)
	1–4	31 (19.9)	125 (80.1)
	5–8	28 (31.8)	60 (68.2)
	≥9	6 (25.0)	18 (75.0)
Residence [n (%)]	Community-dwelling	59 (22.4)	204 (77.6)
	Institution	8 (29.6)	19 (70.4)
Number of regular medications [n (%)]	0	1 (6.7)	14 (93.3)
	1–5	11 (11.6)	84 (88.4)
	6–10	33 (28.9)	81 (71.1)
	11–15	19 (36.5)	33 (63.5)
	≥16	3 (21.4)	11 (78.6)
Number of medicines taken “when necessary”	0	11 (12.4)	78 (87.6)
	1–3	33 (23.7)	106 (76.3)
	4–6	19 (40.4)	28 (59.6)
	≥7	4 (26.7)	11 (73.3)
Polypharmacy [n (%)]^*^	Yes	58 (28.2)	148 (71.8)
	No	9 (10.7)	75 (89.3)
Specialty [n (%)]	Internal medicine	33 (21.0)	124 (79.0)
	Surgery	21 (30.9)	47 (69.1)
	Neurology	11 (18.6)	48 (81.4)
	Other	2 (33.3)	4 (66.7)
Age [y: mean ± SD (range)]		79.2 ± 7.9 (65–94)	76.3 ±7.3 (65–95)
Comorbidities [mean ± SD (range)]		4.9 ± 2.5 (0–12)	3.9 ± 2.6 (0–12)
Number of regular medication [mean ± SD (range)]		9.1 ± 4.0 (0–22)	6.8 ± 4.5 (0–22)
Number of medication used “when needed” [mean ± SD (range)]		2.8 ± 2.2 (0–11)	2.0 ± 2.3 (0–15)

*Information on patients' demographics (age, sex, living arrangement), comorbidities, medication regimen, ADE, drug interactions, and reason for admission was gathered from electronic patient records. Admissions were divided into groups according to different sociodemographic variables for further analysis*.

* *OR 3.3; 95% CI, 1.5–6.9; p = 0.001*.

The causality of ADE and reason for admission was assessed by the multi-disciplinary research team. In addition, causality assessment was conducted with the customized Naranjo scale as a control method. Because the research was conducted retrospectively it was not possible for any single case to receive more than 7 points on the Naranjo assessment scale (e.g., it was not possible to answer the questions “did the reaction appear when placebo was given?” “did the reaction reappear when drug was readministered,” and the answer to the question “are there alternative causes that could on their own have caused the reaction” was consistently “yes”). Accordingly, the Naranjo scale could not be used in this study to indicate causality as “definite.” Therefore, the results from both research team assessment and the Naranjo assessment criteria were divided into three categories; probable, possible and doubtful. Each of the visits was categorized into one of these groups. The assessment criteria of both research team and the customized Naranjo assessment scale are presented in Table 1.

### Statistics

Statistical analysis was carried out using SPSS Statistics 23.0 (SPSS Inc., Chicago, IL, USA). Descriptive statistics were used to describe patient characteristics. Pearson's chi-squared tests (χ2) were used to test the relationship between discontinuous variables. The differences between the sociodemographic data and mean values were examined by *t*-tests or Analysis of variance (ANOVA) with *post-hoc* Tuckey tests. Odds ratio (OR) was used to estimate the value of the association of certain variables and hospitalization and it was presented with its 95% CI. *p* < 0.05 was set as the level of statistical significance in the two sided approach.

## Results

The research data consisted of 290 ED admissions made by 287 patients. Of these admissions, the majority was made to the specialties of internal medicine, neurology, and surgery. The rest of the specialties (dental surgery, ear, nose and throat diseases, ophthalmology, and gynecological diseases) were grouped as “others.” Most admissions were made by community-dwelling patients and only a minority of the patients was from institutionalized care.

The age of the patients varied between 65 and 95 years and the average age was 77 years. For further analysis, patients were divided into three age groups, 65–74, 75–84, and 85–95 years (Table 2). In comparison, the youngest age group had significantly less comorbidities than the older age groups (mean value 3.5 vs. 4.5 and 4.8, *p* = 0.004, respectively). A significant variation between age groups was also detected in the number of regular medications and medicines taken “when necessary.” The average number of regular medicines increased with age; in the youngest age group the number of regular medicines was 6.6, in the middle age group 7.5, and in the oldest age group 8.9 (*p* = 0.012),whereas the number of medicines used “when needed” was 2.0 for the two younger age groups and 3.1 for the oldest (*p* = 0.04). The majority (*n* = 206, 71%) of the patients were affected by polypharmacy. Age was found to increase the likelihood for polypharmacy (*p* < 0.001). Residence, however, did not appear to affect the relative risk for polypharmacy in our study small sample (*p* = 0.268).

Out of 290 admissions, 67 (23.1%) were found “probably” or “possibly” medication-related (*n* = 38 and *n* = 29, respectively) (Table 2). Age, sex, residence, or specialty did not affect the appearance of medication-related admissions (min *p* = 0.077). Polypharmacy, on the other hand, was found to increase the likelihood for medication-related hospital admission (*p* = 0.01).

Multiple ADEs presented in the medication-related admissions (Table 3). Although the distribution of the ADEs was wide “vertigo, falling, and fractures” was identified as the most common group of ADEs in the study sample. The group “others” included single incidences of ADEs such as impaired mobility, sweating, nausea, leukopenia, unconsciousness, swelling, and wounds in skin and oral mucosa. Only 27 (40.3%) ADEs of the found 67 ADEs were detected at the time of admission to the ED.

**Table 3:** **Detected ADEs in the study sample of 290 ED admissions and medications related to them (*n* = 67)**.

**ADE description**	**Number of events, *n* = 67 (%)**	**Causative drug (times involved in ADE)**
Falling, vertigo, fractures	13 (19.4%)	Oxycodone(3), diazepam(3), isosorbide mononitrate(2), memantine(3), levodopa(3), risperidone(2), hydroxyzine(2), isosorbide dinitrate(1), lithium(1), haloperidol(1), temazepam(1), mirtazapine(2), topiramate(1), amitriptyline(1), tramadol(1), tiotropium(1), rivastigmin(1), nifedipine(1), codein(1), glyseryl trinitrate(1), tizanidine(1)
Bleeding	8 (12.0%)	Warfarin(5), prednisolone(1), acetylsalicylic acid(1), clopidogrel(1), enoxaparin(1), venlafaxine(1)
ADR or infection after cytostatics treatment	8 (12.0%)	Docetaxel(2), fluorouracil(1), azacitidine(1), panitumumab(1), tamoxifen(1), rituximab(1), cyclophosphamide(1), epirubicin(1), doxorubicin(1), vincristine(1), methotrexate(1), temozolomide(1)
Disorientation, delirium, memory loss	6 (8,9%)	Fentanyl(1), Buprenorphine(1), clonazepam(1), carbamazepine(1), zopiclone(1), amitriptyline(1), diazepam(1), chlordiazepoxide(1), tramadol(1), oxycodone(1)
Constipation, occlusion	6 (8.9%)	Buprenorphine(2), risperidone(2), ispagula extract(1), loperamide(1), amitriptyline(1), chlordiazepoxide(1), codein(1), haloperidol(1), quetiapine(1), duloxetine(1), oxycodone(1), memantine(1)
Decrease in general condition	6 (8.9%)	Buprenorphine(1), donepezil(1), digoxin(1), oxycodone(1), pregabalin(1), escitalopram(1), quetiapine(1), prednisolone(1), carbamazepine(1), duloxetine (1), isosorbide mononitrate(1), ramipril(1)
Infection after immunosuppressive treatment	5 (7.5%)	Methylprednisolone(1), Prednisolone(3), hydrocortisone(1)
Arrhythmias	4 (6.0%)	Donepezil(1), solifenazin(1), digoxin(1), verapamil(1), bisoprolol(1), furosemide(1)
Convulsion	3 (4.5%)	Citalopram(1), quetiapine(1), duloxetine(1), fesoterodine(1), tiotropium(1), teophyllin(1), risperidone(1), mirtazapine(1)
Other	8 (11.9%)	Calcium(1), iron(1), scopolamine(1), enalapril(1), clozapine(1), levodopa(1), lerchanidipin(1), azathioprine(1), furosemide(1), metformin(1)

**Figure 2:**
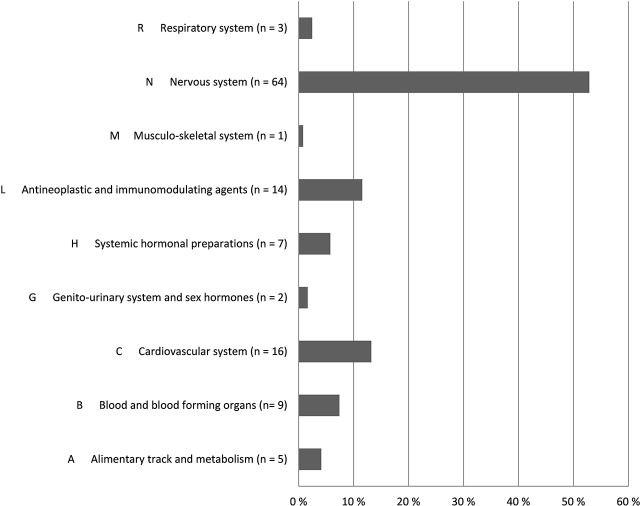
**Distribution of medicines involved in ADEs by ATC codes (*n* = 121) according to research team and customized Naranjo scale**.

Altogether 121 drugs from 9 different ATC groups (Anatomical Therapeutic Chemical Classification System) were involved in the ADEs (Figure 2). The majority (64, 52.9%) of the drugs were N class medication (nervous system) (Table 4). Medicines from L (antineoplastic and immunomodulating agents) and C (Cardiovascular disease) classes were also frequently associated with found ADEs. Together these three groups covered 76% of all medicines causing ADEs in the study sample. The majority of the ADEs resulted from additive effect of more than 1 medicine. Furthermore, medicines from the different ATC groups could be involved in the same ADE.

**Table 4:** **The distribution of ATC-code N class medicines involved in found ADEs (*n* = 64)**.

**ATC code** **N nervous system**	**No. of medications** ***n* = 121 (%)**
N02A Opioid drugs	15 (12.4%)
N03A Antiepileptic drugs	5 (4.1%)
N04 Antiparkinsonian drugs	4 (3.3%)
N05A Antipsychotic drugs	12 (9.9%)
N05B Anxiolytic drugs	7 (5.8%)
N05C Hypnosis and sedative drugs	2 (1.7%)
N06A Antidepressant drugs	11 (9.1%)
N06CA Antidepressants in combination with psycholeptics	1 (0.8%)
N06D Dementia drugs	7 (5.8%)

All the included study admissions were assessed by the customized Naranjo scale alongside the research team's causality assessment. The purpose of this was to determine whether there was any differences in detection of ADEs between the two methods. To make the comparison easier, the results from both assessment methods were categorized into three similar groups (probable, possible, and doubtful).

**Figure 3:**
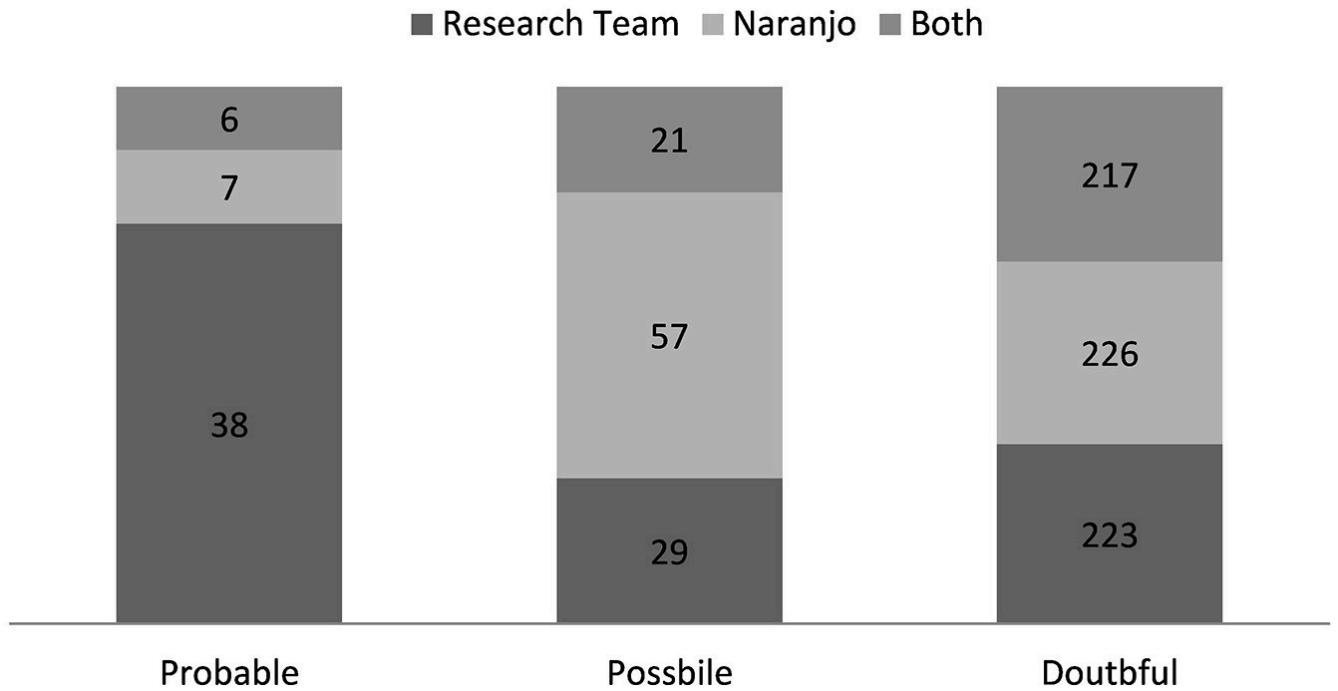
**Causality assessment; comparison the causality assessment of research team and the customized Naranjo scale (*n* = 290)**.

In total, 223 and 226 admissions were categorized “doubtful” by research team and customized Naranjo scale, respectfully (Figure 3). 217 of these admissions were categorized “doubtful” by both of the assessment methods. In the two other groups the distribution was larger. The customized Naranjo scale placed the majority of the remaining admissions as “possible,” whereas the research team categorized admissions more evenly between “probable” and “possible.” Only six admissions were categorized by both assessment methods as “probable.”

## Discussion

To our knowledge, there are no studies published on the prevalence of geriatric hospitalizations due to ADEs in Finland. This research therefore provides valuable information on national challenges faced when medicating the elderly. International studies have shown that ADEs cause approximately 5–50% of all geriatric hospital admissions with large deviations mainly resulting from definition differences (Budnitz et al., 2007; Pascale et al., 2009; Alwahassi et al., 2014; Davies and O'Mahony, 2015). In this study sample, ADEs were discovered to cause 23.1% of geriatric ED hospitalizations and thereby placing the prevalence significantly higher than the 10% median of ADR-related hospitalizations in recent review (Alwahassi et al., 2014).

Out of the 290 patients included in this study, 157 were treated in the specialty of internal medicine whereas the number of included surgical patients was 68. The expected number of included patients in internal medicine and surgery were 122 and 105, respectively. In addition, the number of excluded patients in internal medicine and surgery were 18 and 75 and the expected number of excluded patients 47 and 40, respectively. Accordingly, fewer patients were included and far less patients excluded from surgery than expected. As all the exclusions made were due to lacking information on medication regimen, it was concluded that medication was not reconciled in a large fraction of surgical patients in ED during admission posing a significant threat to overall patient safety during treatment. Consequently, medication-related admissions appeared more frequently in surgical patients than in internal or neurological patients. The reasons for the inadequate documentation of medication regimen could not be established but the findings emphasize the need for consistent medication reconciliation in all specialties.

There was no statistically significant difference in the occurrence of medication-related hospitalization between age groups, sex, specialties, or residence in our small study sample. Polypharmacy, on the other hand, was found to increase the risk of medication-related admissions. The most common group of ADEs (20.9%) was “vertigo, falling, and fractures.” However, the difference between this and the other ADE groups was small indicating fairly heterogeneous expression of ADEs. The overall results on factors related to ADEs suggest that screening for patients with specific demographics or symptoms would not result in improved detection of ADEs in the patient population. Furthermore, recognizing patients with ADEs on larger scale would require more effective ways of data-mining in addition to more accurately focused resources.

In this study, we found medicines from the ATC N class (nervous system) to cover most of the medicines (52.9%) involved in ADEs. Furthermore, the N class medicines took part in all ADE groups except “infection after immunosuppression” and “ADR or infection caused by cytostatics.” Previously, drugs from this category have been associated with falling, anticholinergic symptoms, delirium, cerebrovascular events, parkinsonism, and oversedation of geriatric patients (Ballard et al., 2006; Woolcott et al., 2009; Clegg and Young, 2011; Gillespie et al., 2012; Hovstadius et al., 2014; Palmer et al., 2015; Salahudeen et al., 2015). In our study, the most frequent subgroups leading to ADEs were opioids, antipsychotics and antidepressants. Adverse outcomes were especially associated with simultaneous use of several drugs from these groups. In this small study, however, we could not say whether the ADE was preventable or not.

When compared with each other, the assessment by research team and the customized Naranjo scale assessed a near equal amount of admissions “doubtful.” Nevertheless, much variation was discovered between the groups “probable” and “possible.” The customized Naranjo scale was inclined to assign more admissions to “possible” whereas, research team categorized more admissions as “probable.” Most of the variation can be explained by huge contrast in method flexibleness. The research team could regard a variety of affecting variables in the decision making whereas the Naranjo scale is based on fixed questions. The fixed nature of structured assessment methods has been considered one of its best assets as the result shouldn't vary between different assessors. However, structured methods are often insufficient when assessing cases in complex context, e.g., polypharmacy or multimorbidity. Thus, in a clinical setting the ability to adapt has greater significance in distinguishing the “probable” from the “possible.”

There are some limitations in this study. The take of 290 patients is small as well as the number of patients hospitalized due to ADEs. This must be considered when interpreting the results. Relatively large number of surgical patients was excluded as a result of missing medication information. Due to the retrospective nature of the study it was not possible to gather data concerning some variables, e.g., frailty, that could have affected the outcome. This study, however, provides valuable information on admissions caused by ADE in the aged population.

Most of the current efforts to detect ADEs are centered on voluntary reporting systems, retrospective manual tracking of errors, and different chart audits. Pharmacovigilance actions and risk management—although prospectively included in modern drug development—are however methodologically based on follow-up and focused on signal detection, rather than prevention and clinical management of ADEs. In the end, with such approaches, only 5–10% of ADEs are ever reported and, of those, up to 95% actually do not cause any harm to the patient, clearly indicating that these traditional methods are insensitive, ineffective and expensive (Naessens et al., 2009; Classen et al., 2011; Kennerly et al., 2014). The majority of ADEs could, however, be predicted and avoided beforehand. Thus, it is evident that there is a great need for more effective approaches to identify and prevent ADEs in order to reduce harm.

## Author contributions

OL conducted data collection. OL, SS, RB, and MT were involved in data analyses and interpretation of the results. All authors participated in research design, contributed to the writing of the manuscript and approved the final manuscript.

## Funding

This study was funded by the Oulu University Hospital Grant (K10754, Turpeinen).

### Conflict of interest statement

The authors declare that the research was conducted in the absence of any commercial or financial relationships that could be construed as a potential conflict of interest.
